# Maladaptation in feral and domesticated animals

**DOI:** 10.1111/eva.12784

**Published:** 2019-03-18

**Authors:** Eben Gering, Darren Incorvaia, Rie Henriksen, Dominic Wright, Thomas Getty

**Affiliations:** ^1^ Department of Integrative Biology and Ecology, Evolutionary Biology, and Behavior Program Michigan State University East Lansing Michigan; ^2^ IIFM Biology and AVIAN Behavioural Genomics and Physiology Group Linköping University Sweden

**Keywords:** adaptation, artificial selection, domestication, feralization, invasion, maladaptation

## Abstract

Selection regimes and population structures can be powerfully changed by domestication and feralization, and these changes can modulate animal fitness in both captive and natural environments. In this review, we synthesize recent studies of these two processes and consider their impacts on organismal and population fitness. Domestication and feralization offer multiple windows into the forms and mechanisms of maladaptation. Firstly, domestic and feral organisms that exhibit suboptimal traits or fitness allow us to identify their underlying causes within tractable research systems. This has facilitated significant progress in our general understandings of genotype–phenotype relationships, fitness trade‐offs, and the roles of population structure and artificial selection in shaping domestic and formerly domestic organisms. Additionally, feralization of artificially selected gene variants and organisms can reveal or produce maladaptation in other inhabitants of an invaded biotic community. In these instances, feral animals often show similar fitness advantages to other invasive species, but they are also unique in their capacities to modify natural ecosystems through introductions of artificially selected traits. We conclude with a brief consideration of how emerging technologies such as genome editing could change the tempos, trajectories, and ecological consequences of both domestication and feralization. In addition to providing basic evolutionary insights, our growing understanding of mechanisms through which artificial selection can modulate fitness has diverse and important applications—from enhancing the welfare, sustainability, and efficiency of agroindustry, to mitigating biotic invasions.

## 1 INTRODUCTION

Darwin utilized diverse case studies of domesticated species to illustrate how selection drives phenotypic change (Darwin, 1859; Darwin, 1868). He also emphasized that domestication is unique in producing these changes via both “methodical” artificial selection for human‐desired traits and “unconscious” selection for other traits that evolve unintentionally via captive propagation in unnatural environments (Driscoll, Macdonald, & O'Brien, 2009; Tillotson, Barnett, Bhuthimethee, Koehler, & Quinn, 2019). Both of these components of the domestication process are often assumed to leave taxa maladapted for life outside of captivity (e.g., Baskett & Waples, 2013) and to constrain their potential for further adaptive evolution (e.g., Marsden et al., 2016; Schubert et al., 2014). Nonetheless, animal breeders continue to improve many aspects of performance in captive settings, and diverse populations of formerly domesticated taxa (e.g., feral dogs, cats, and pigs) are thriving around the globe. We suggest that the mechanisms that permit or hinder this success merit further investigation, since cultivated, urbanized, and wild ecosystems are increasingly interconnected, and because rapid evolutionary changes can occur in each setting (Sarrazin & Lecomte, 2016; Turcotte, Araki, Karp, Poveda, & Whitehead, 2017). At a practical level, characterizing the evolution and impacts of domestic and feral taxa is an important step toward evolutionarily informed management of zoonotic diseases, ecosystem functions, and agricultural sustainability, efficiency, and welfare. Finally, at a more basic level, domestication and feralization each offer unique opportunities to study evolutionary responses to novel and changing environments in tractable model systems (Table 1). Biological insights from these organisms may additionally shed light on the evolution our own species, which is proposed to have “self‐domesticated” and exhibits many demographic and environmental similarities to domesticated nonhumans (Burkart et al., 2014).

**Table 1:** Divergent selection regimes of wild/feral and domesticated populations

	“Wild” environment^a^	Domestic environment	Targeted traits
Sexual competition	Mate competition and choice among many (syntopic) partners	Mate competition is reduced or eliminated (e.g., via studbooks, pedigrees, artificial insemination)	Sexual characteristics, behavior, reproductive biology
	Operational sex ratio shaped by the local environment	Operational sex ratio optimized for production	
	Sexual signaling and mate searching in complex environments	Sexual signaling and searching in homogeneous environments	
Social interactions	Lower population densities	Higher population densities	Aggression, parental investment, morphology, life history, cognition, sensory systems
	Fluid age structures and social groups	Human‐controlled age structures and social groups, restricted and/or augmented parental care	
	Self‐directed territoriality	Human‐structured territories	
	Wild‐type behaviors	Breeding for docility	
Diet	Variable diet determined by local environment	Abundant, homogenous, and enriched food supply	Metabolism, digestion, microbiome, foraging behavior, life history
Natural enemies	Predators and competitors	Protection from predators and reduced competition	Immunogenetics, microbiome, behavior
	Diversified pathogen transmission networks	Localized pathogen outbreaks in homogeneous host communities	
	Ecological modulation of immunity and exposure	Human‐mitigated disease risks and costs (e.g., vaccines, antibiotics, and probiotics)	
Abiotic environment	Heterogeneous and fluctuating environments	Stabilized microenvironments	Morphology, physiology, behavior

a Selection pressures in feral habitats are often broadly similar to those of ancestral wild environments, yet may also differ due to dispersal beyond the native range, anthropogenic disturbances, and/or other environmental changes that postdate domestication.

In this following review, we summarize ideas and case studies that illustrate how maladaptation arises during, is illuminated by, and/or emerges from domestication and feralization. While these themes have been explored in earlier reviews, prior syntheses have chiefly focused on the consequences of maladaptation for animal production (e.g., Mignon‐Grasteau et al., 2005; Price, 1999) and have also predated new and informative work catalyzed by genome sequencing technologies. Our synthesis of current knowledge yields both intuitive and surprising conclusions about the impacts of artificial selection on organisms, populations, and communities, including (a) domestication‐related fitness trade‐offs, relaxed natural selection, and genetic load can incur fitness costs in both captive and wild environments, (b) feralization can expose both costs and benefits of domestication histories, as well as revealing “standing” maladaptation within other members of invaded communities, and (c) through diverse and complex effects on connected ecosystems, both feral invasions and domestication practices can also produce maladaptation in wild organisms. Understanding the diverse mechanisms by which artificial selection histories modulate fitness has both conceptual and applied significance. Attenuating maladaptation in production settings bolsters agroindustrial efficiency and sustainability, whereas limiting adaptation in feralizing taxa can curtail their roles in biotic invasion.

## 2 MALADAPTATION UNDER ARTIFICIAL SELECTION

Crespi, (2000) recently synthesized key concepts and challenges surrounding the study of maladaptation. Maladaptation takes many forms (as outlined in Table 2) and can be investigated at the levels of individuals or populations, as a standing pattern or evolutionary process, and as a factor that limits either absolute fitness, or fitness relative to some other reference individual, population, and/or timepoint. These issues also apply to special cases of maladaptation that involve artificial selection, which we focus on in this review. Humans have domesticated diverse animal taxa for equally diverse purposes—including food, labor, fiber, and companionship. Within captivity, these taxa typically show higher relative fitness than wild counterparts—becoming more abundant and widespread than their source populations through human facilitation. In cases such as cattle (Taberlet et al., 2008) and horses (Gaunitz et al., 2018), domestication has even allowed species to survive extinctions of conspecifics in natural settings. Thus, ongoing human facilitation has proven highly adaptive, in terms of both relative and absolute fitness, for many domesticated taxa.

**Table 2:** Maladaptation mechanisms in domestication and/or feralization contexts

Maladaptation mechanism(s)	Instance(s) in domestic and feral animals
Suboptimal traits result from genetic drift, gene flow, or mutation	Genomic data indicate domestication‐related bottlenecks have reduced the efficiency of selection in several taxa (Chen, Ni et al., 2018). In many domestic species, inbreeding depression has reduced viability and/or increased disease susceptibility (Peripolli et al., 2017)
Suboptimal trait variance reduces population fitness	Phenotypic variation is often intentionally reduced within, and enhanced among, specialized breeds selected for divergent environments and/or purposes. Behavioral variation can also evolve rapidly as a by‐product of captivity. Laboratory mice, for example, exhibit more variable (and also reduced) responsiveness to predators compared to wild populations; these changes are predicted to reduce fitness during feralization (McPhee, 2010)
Accumulation of mutations reduces fitness	Observed excesses of deleterious mutations have been described as a cost of domestication in several species including dogs (Cruz, Vilà, & Webster, 2008; Marsden et al., 2016) and horses (Schubert et al., 2014)
Changing environments cause trait–environment mismatch	Both domestication and feralization bring rapid environmental changes (Figure 1, Table 1). For example, enriched diets contribute to metabolic disease and can hinder cardiovascular, skeletal, and immunological performance in captivity (e.g., Burns et al., 2015). Adaptations to captivity, such as antibiotic resistant microbiomes, can also persist through feralization (Ferrario et al., 2017) and may impact fitness in the wild
Changing environments alter fitness differentials of traits	Genomic data suggest relaxed natural selection is pervasive during domestication (e.g., McPhee, 2010, Björnerfeldt et al., 2006, Chen, Zhang et al., 2018). Resulting changes in domesticated gene pools will likely impact selection differentials among animals recolonizing the wild
Environmental degradation reduces fitness	Environmental changes impact the suitability of global habitats for domesticated and feral animals (Craine, Elmore, Olson, & Tolleson, 2010; Thornton, Steeg, Notenbaert, & Herrero, 2009), and can also affect the quality and quantity of animal feed (e.g., Battilani et al., 2016). For example, anthropogenically driven droughts and wildfires (Wehner, Arnold, Knutson, Kunkel, & LeGrande, 2017) are causing die‐offs in both feral and domestic animals of the American west
Fitness is limited by co‐evolving organisms	Domestication has driven the evolution and spread of virulent pathogens (Read et al., 2015), antibiotic resistant microbes (Van den Bogaard, London, Driessen, & Stobberingh, 2001), and sexually transmitted tumors (Murchison et al., 2014) that can reduce the health and survival of domesticated taxa
Fitness is reduced by feedback between environment and trait variance	Domestication can alter the variance of many traits that are potentially involved in eco‐evolutionary dynamics (e.g., behavior and life history; Price, 2002). However, feedbacks between the environment, trait evolution, and fitness have not been well studied
Density and/or per capita resource consumption degrade local environments	Density‐dependent population growth has been documented in many feral and domesticated animals (e.g., Choquenot, 1991). For example, the classic “tragedy of the commons” scenario describes how overexploiting shared resources (here, pastures) can decrease the absolute fitness of domesticated populations

At the same time, artificial selection is usually assumed to leave animals unfit for survival and reproduction outside the confines of captivity. This presumed maladaptation is implicitly conceptualized in terms of the imagined fitness of individuals or populations inhabiting ancestral (wild) and/or future (feral) habitats, though such fitness is rarely studied formally. Limited research in this area, stemming chiefly from fish models, shows that even a single generation in captivity can radically alter heritable phenotypes (Christie, Marine, Fox, French, & Blouin, 2016; Fraser et al., 2019). Further, this brief cultivation can quickly and powerfully reduce individual fitness (relative to undomesticated counterparts) when captive animals are reintroduced to the wild (Christie, Ford, & Blouin, 2014). As we discuss in section V, admixture can also negatively impact the fitness of wild populations that interbreed with feral or captive relatives (Castellani et al., 2018; McGinnity et al., 2003; Skaala et al., 2012). On the other hand, the recent exponential growth of many feral populations, particularly those invading ecosystems which are already occupied by low densities of wild conspecifics (e.g., feralizing chickens; Gering, Johnsson, Willis, Getty, & Wright, 2015), shows that “legacy” effects of artificial selection can vary widely among feralization episodes. To incorporate evolutionary planning into the management of captive and feral populations, it is therefore important to ascertain the sources of this variability.

In addition to modulating fitness within noncaptive habitats, domestication practices can attenuate fitness within captivity. The mechanisms behind these fitness declines, which we describe in more detail below, include antagonism between artificial and natural selection, effects of captive breeding practices on standing genetic variation, and environmental changes that negatively impact the quality of captive environments. These sources of maladaptation can also interact and intensify with passing time (i.e., generations) in captivity—as seen in the escalating infertility and disease susceptibility of various domesticated breeds. Declining fitness within cultivated settings presents ongoing challenges for animal breeders wishing to maintain or improve animal performance. Thus, in addition to advancing our general understanding of evolution, studies of maladaptation in captive settings can abet management of the narrow array of animal species that have most profoundly shaped human civilization and evolution (Diamond, 2002).

## 3 MALADAPTIVE TRADE‐OFFS UNDER ARTIFICIAL SELECTION

Maladaptive trade‐offs can occur whenever artificial selection promotes animal traits at the expense of survival and/or reproduction. While affected individuals or populations can still exhibit reproductive success or positive growth rates, fitness under maladaptive trade‐offs is also reduced relative to idealized references that are exempt from the trade‐offs. Comparing the realized and potential fitness of animals subject to trade‐offs has both conceptual and practical benefits, because adjustments to breeding programs, enhancement of captive environments, and/or genome editing can feasibly reduce or eliminate trade‐offs that otherwise constrain absolute fitness. It is therefore both interesting and useful to examine these trade‐offs' underlying causes.

Maladaptive trade‐offs can arise from both pleiotropy and genetic correlation between fitness‐related and artificially selected traits. For many traits of interest, it is not yet possible to distinguish between these two sources of trade‐offs; doing so requires elucidation of the genetic architectures of focal traits, including their covariance with other fitness‐related phenotypes. One example of pleiotropic maladaptation is found in bulldogs, which were bred to have short and stout stature that renders them virtually incapable of effective copulation (Pedersen, Pooch, & Liu, 2016). These animals now rely on artificial insemination or mechanical assistance to reproduce; they are therefore maladapted for self‐propagation. At present, however, these organisms still retain high absolute fitness due to popularity with humans and facilitation by breeders.

Less extreme pleiotropic fitness trade‐offs are probably common among other artificially selected animals, given that trait elaboration for production purposes or human fancy will often be opposed by natural selection (Rauw, Kanis, Noordhuizen‐Stassen, & Grommers, 1998). In broiler chickens, for example, skeletal, reproductive, metabolic, and circulatory disorders result in mortality rates as high as 20% per flock; these deleterious effects on absolute fitness are also understood to be pleiotropic consequences of selection for accelerated growth (Balog, 2003). Genetic mapping studies have further suggested that pleiotropy modulates other quantitative behavioral, morphological, and life‐history traits of domesticated chickens (Wright et al., 2010). However, the mapped regions that impact these traits may contain tightly linked and interacting mutations (Wright, 2015), the form(s) of artificial selection that produced them is not known, and their connection to absolute or relative fitness requires further investigation.

Pleiotropy is also suggested to have contributed to the “domestication syndrome” that Darwin identified in many domesticated vertebrates. These animals show strikingly similar, evolutionarily derived distinctions from their wild relatives in behavior, body size, skeletal morphology, coloration, brain structure, development, and endocrinology. Comparative studies of domesticated genomes and developmental programs have supported the possibility that these correlated changes emerged from artificial selection and pleiotropy. Specifically, domesticated phenotypes may reflect changes in the orchestration of diversely fated cells that originate within embryonic neural crests (Wilkins, Wrangham, & Fitch, 2014). This idea is supported by well‐known studies of captive‐reared foxes, in which researchers discovered that morphological, behavioral, and physiological aspects of domestication syndromes can be experimentally recapitulated by selecting only on animal tameness (Belyaev, 1979; Trut, Oskina, & Kharlamova, 2009). Reduced fear of humans is a distinguishing feature of many domesticated taxa, and this was likely either artificially selected by early humans or naturally selected within human commensals during the earliest stages of domestication (Price, 1999). This positive selection for tameness, and resultant changes in developmentally linked traits, might therefore explain how diverse animal taxa acquired domestication syndromes independently (Sánchez‐Villagra, Geiger, & Schneider, 2016).

Recent experimental studies imposing artificial selection for tameness on wild animals have further supported its potential role in the production of domestication syndromes. In wild Red Junglefowl, which are conspecific with domesticated chickens, selection for tameness rapidly generates heritable shifts toward “domestic‐like” growth, metabolism, and behavior that may be under genetic and epigenetic control (Agnvall, Bélteky, Katajamaa, & Jensen, 2018). Similarly, over just a 10‐year period, a domestication syndrome involving amelanic fur patches and reduced head length emerged unexpectedly within semi‐captive wild mice selected indirectly for tameness through frequent human handling (Geiger, Sánchez‐Villagra, & Lindholm, 2018). These recent experiments show how both “conscious” artificial selection and self‐domestication could feasibly have produced the domestication syndromes Darwin first observed in modern domesticated animals.

At the mechanistic level, genome scans of domesticated taxa also indicate that domestication syndromes may have arisen from selection on neural crest development. Rapid evolutionary changes at loci coordinating neural crest cell fates have been found in several taxa that were domesticated for diverse human utilities, including village dogs (Pendleton et al., 2018), housecats (Montague et al., 2014), and horses (Librado et al., 2017). These parallel phenotypic and genomic changes raise the question of whether these animals' ancestors were uniquely predisposed for domestication. In this case, other taxa would then be comparatively maladapted for domestication and/or self‐domestication via human commensalism. Given a rapidly increasing human presence throughout global ecosystems, and our species' outsized role in ongoing extinctions of native wildlife, such differences may be a crucial determinant of future species persistence (Teletchea, 2017). Of more immediate significance to animal breeders and human health, there is also evidence that certain genetic maladies in humans, and perhaps in other taxa as well, arose through pleiotropic effects of changes in the neural crest developmental pathway produced by domestication (of animals) or self‐domestication (of humans; e.g., Bolande, 1974, Benítez‐Burraco, Lattanzi, & Murphy, 2016, Benítez‐Burraco, Pietro, Barba, & Lattanzi, 2017).

Maladaptive trade‐offs can also result from physical linkage or epistasis between the genomic loci targeted by artificial selection and other genes that modify absolute and/or relative fitness of individuals or populations (Crespi, 2000), and via gene × environment interactions. Prior work suggests these factors may have limited influence over many domestication‐related traits, which have shown a relatively simple genetic basis and also consistent expression among cultivated environments. These features of known “domestication genes” would thus limit fitness modulation through epistasis or gene–environment interactions (Wright et al., 2010). Still, the genetic basis of many domestication‐related traits remains unknown, and a subset are also known to involve genetic correlations that could impose evolutionary constraints (e.g., Le Rouzic, Álvarez‐Castro, & Carlborg, 2008, Larson et al., 2014). Returning to man's best, albeit maladapted friend (the dog), selection on body mass and behavior has driven divergence among breeds in genetically correlated and heritable components of the “pace of life” (Careau, Réale, Humphries, & Thomas, 2010). While human fancy remains the key determinant of breed fitness in captivity, variation in pace of life is predicted to have context‐dependent effects during feralization, potentially favoring differently adapted breeds (i.e., paces of life) in environments with high versus low resource distributions (Dammhahn, Dingemanse, Niemelä, & Réale, 2018). Genetic linkage between heritable components of complex animal phenotypes might also be altered during feralization, but this has not been well studied. Closing this knowledge gap will be important for assessing how genome architecture and genome reorganization ultimately contribute to feralization outcomes.

## 4 RELAXATION OF NATURAL SELECTION UNDER ARTIFICIAL SELECTION

Relaxed natural selection in captivity can have important evolutionary consequences for domesticated organisms. Captive environments are often enriched in numerous ways that increase animal health and productivity by reducing malnourishment, stress, and disease; these include food provisioning, climate control, predator exclusion, and veterinary care. Both theory and molecular data suggest that these relaxations of selection pressures can promote standing and de novo genetic variation in captivity. In domesticated geese, for example, relaxed selection for flight capability is proposed to explain elevated accumulations of nonsynonymous mutations within oxygen transport genes (Wang et al., 2017). Domesticated yaks also show elevated rates of amino acid substitutions in mitochondrially encoded genes. Presumably, this difference reflects the stronger influence of selection for metabolic efficiency in wild yaks, which inhabit cold and hypoxic high altitudes of the Qinghai–Tibetan Plateau. While climate‐associated divergence in metabolic genes is not ubiquitous among domesticated lineages (Moray, Lanfear, & Bromham, 2014), it has also been observed in dogs (Björnerfeldt, Webster, & Vilà, 2006) and in chickens (Zhao et al., 2016).

In addition to relaxing physical selection pressures, domestication can curtail both ecological and social selection regimes. For example, the practice of culling mature sheep and goats reduces aggressive dominance rivalries between older males. This reduction in social competition may have driven diminutions of sexual dimorphism in domesticated caprine breeds (Zohary, Tchernov, & Horwitz, 1998). Restoration of social competition could also explain recent positive selection for larger horns in semi‐feral caprine populations (Pan et al., 2018). Similarly, recent rapid evolution at genomic loci controlling social behavior in feral chickens (Johnsson et al., 2016) suggests that domestication may leave animals maladapted for the social challenges they encounter in early stages of feralization. Lastly, domestication can also relax many forms of ecological selection that exert strong purifying, positive, or fluctuating selection in nature. For instance, captive‐propagated mice exhibit reduced responsivity to cues of predator presence (Blanchard et al., 1998), and minor bill abnormalities in feral pigeons render them less adept at removing fitness‐reducing ectoparasites by preening (Clayton, Lee, Tompkins, & Brodie, 1999).

Both the literature and casual observations offer many additional examples of evolutionary losses of wild‐adapted traits in captive populations. Collectively, the chosen case studies above illustrate how relaxed natural selection in the enhanced and protected environments that humans provide for our animals can lead to the elimination of natural defenses from enemies, social competitors, and physiological stress. It is worth noting, however, that relaxed natural selection can also facilitate the evolution of adaptive phenotypic plasticity (Hunt et al., 2011) and may permit organisms to reach higher fitness peaks by reducing the ruggedness of adaptive landscapes (Svensson & Calsbeek, 2012). Thus, artificially relaxed natural selection can have complex and opposing effects on the fitness of both domestic and feral taxa.

Intriguingly, self‐domestication and attendant relaxation of selection may also have shaped the recent evolution of our own species' cognition, language capability, development, physiology, and life history (Deacon, 2010; Kuhlwilm & Boeckx, 2018; Theofanopoulou et al., 2017). For example, heritable deleterious traits affecting visual and craniofacial development are more prevalent in civilized populations—suggesting these comparatively enriched environments may buffer purifying selection (Post, 1971). These similarities between humans and other domesticated organisms make our beasts of burden valuable tools for the investigation of recent human evolution, including self‐domestication's potential effects on maladaptive human traits such as genetic disease (Boyko, 2011; Johnsson, Williams, Jensen, & Wright, 2016; Persson, Wright, Roth, Batakis, & Jensen, 2016).

## 5 EFFECTS OF POPULATION HISTORIES ON STANDING GENETIC VARIATION

The final class of maladaptation mechanisms we consider in this review arises from the unusual population structures of domesticated and feral organisms. Both founder effects and breeding designs can winnow genetic variation from a captive population (Wilkinson & Wiener, 2018). As a result, most domesticated species have lower genetic diversity than their wild relatives do (e.g., Skaala, Hoyheim, Glover, & Dahle, 2004), though many breeds are now managed to maximize their divergence and/or variability (Groeneveld et al., 2010; Muir et al., 2008; Zeder, 2006). Reductions in standing genetic variation can occur at each stage of domestication. Genetic bottlenecks, for example, can occur both during accession into captivity and through the serial dispersal of captive animals among farms, regions, or continents. Additionally, both intentional and stochastic effects of nonrandom breeding designs can further winnow genetic variability—with the strength of these effects dependent on population sizes, structures, and breeding designs (Dekkers, Gibson, Bijma, & Arendonk, 2004).

The erosion of genetic variation can lead to maladaptation in two well‐described ways. First, by limiting the raw material available to selection, it hinders potential future adaptive responses to both artificial and natural selection pressures. Next, bottlenecks and inbreeding can also lead to accumulations of deleterious variants within domesticated genomes; this genetic load can directly reduce population fitness (Marsden et al., 2016). Some features of domestication and feralization, however, can also bolster genetic diversity. These include gene flow between domestic or recently feral animals and related wild organisms and/or interbreeding among genetically divergent breeds. Genetic data are now revealing that such admixture events were far more common in domestication histories than previously appreciated (e.g., Eriksson et al., 2008, Anderson et al., 2009, Larson et al., 2014, Vickrey et al., 2018). Demographic factors can also ameliorate the erosion of genetic variation, which may curtail maladaptive consequences of bottlenecks. Australia's feral rabbits, for instance, are attributed to a single founding episode, yet maintain surprisingly high diversity and fitness due to rapid population expansion—from dozens of individuals to millions in just a decade (Zenger, Richardson, & Vachot‐Griffin, 2003). De novo variation, such as structural rearrangements of genomes, can also arise within captive or feral populations and capacitate adaptive evolution (e.g., Guo et al., 2016). These events are more challenging to detect and consequently less well studied.

## 6 FERALIZATION CAN REVEAL MALADAPTATION IN BOTH DOMESTIC AND WILD ANIMALS

Both the feralization of domestic organisms and the introgression of artificially selected gene variants can shed light on multiple forms of maladaptation. For example, introgression of artificially selected gene variants from domesticated taxa can illuminate prior limits on the relative fitness of wild recipient populations. For example, artificially selected coloration phenotypes may have been positively selected in both wild dog–wolf hybrids and feral pigeons (Anderson et al., 2009; Vickrey et al., 2018). And in wild Alpine Ibex, a gene variant originating from domesticated goats shows a signature of a recent positive selection, which has significantly diversified this wild population's immunogenetic variability. This positive selection on a domesticated gene may subsequently have given rise to frequency‐dependent balancing selection for domestic and wild‐type gene variants (Grossen, Keller, Biebach, & Croll, 2014).

Many domesticated or recently feral populations have also acquired wild‐type gene variants through admixture and positive selection. In these cases, artificially selected populations were thus either imperfectly adapted to their local environments or became so in the immediate wake of introgression events. For example, American populations of European honeybees recently acquired elevated hive defense behavior through admixture with conspecifics that were recently introduced from Africa. Today, a majority of gene variants in feral American honeybees are now of African (i.e., wild‐adapted) origin (Byatt, Chapman, Latty, & Oldroyd, 2016). On an interesting tangent, this introgression may ultimately have reduced the absolute fitness of captive domesticated honeybees via legal restrictions, logistical difficulties, and diminished enthusiasm surrounding apiculture that ensued the evolution of colony‐level aggression. Finally, there is also evidence that recombination between artificially selected and wild‐type alleles may abet the fitness of captive and/or wild populations. For example, genomic variants favored by selection in the feral chickens of Kauai Island appear to have originated from both domesticated and Red Junglefowl gene pools (Johnsson et al., 2016). It would be very interesting to learn whether these selected variants modulate fitness additively or epistatically, as this will reveal how immigration events modify feral fitness landscapes.

The above examples show how introgression can attenuate maladaptation (i.e., increase fitness) in recipient feral or domestic populations. However, admixture and hybridization can also lead to fitness declines in wild populations (Laikre, Schwartz, Waples, Ryman, & GeM Working Group, 2010). This has been best studied in cases of farmed and native fish whose interbreeding reduces the growth, productivity, and lifetime fitness of wild populations (e.g., Fraser, Minto, Calvert, Eddington, & Hutchings, 2010, Glover et al., 2017). These effects can be environment‐dependent (Vandersteen, Biro, Harris, & Devlin, 2012) and can also be transient when selection efficiently purges immigrant gene variants that are locally maladaptive (Baskett & Waples, 2013). Nonetheless, recent theoretical work also shows how maladaptation can persist within recipient populations long after introgression episodes (Tufto, 2017), with the severity and duration of this depending on the alignment of selection regimes of admixing captive and wild source populations.

**Figure 1:**
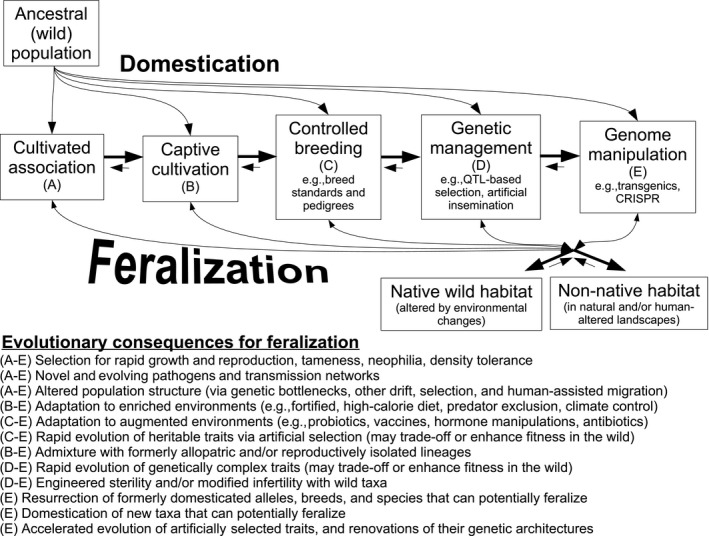
Stages of domestication and their influences on feralization. Captive propagation can begin at any stage along a continuum of domestication practices; these practices also have different influences on the capacities of cultivated populations to recolonize the wild and/or interbreed with free‐living relatives. The accompanying review article considers how such feralization and interbreeding contributes to, and illuminates processes of maladaptation. Note that genome manipulations, which have only recently become possible, will likely have profound and unique effects on both domestication and feralization

With or without introgression, it is noteworthy that feral animals are often tremendously successful at invading non‐native ecosystems. This is best illustrated by the explosive growth of Australia's rabbit populations, which revealed the historical availability of a rich and unoccupied niche spanning most of the continent (Zenger et al., 2003). Many other feral animals (e.g., dogs, cats, and pigs) also thrive in geographical regions that lie well outside the ranges of their ancestral sources. In human‐dominated environments, these cases may partly reflect enduring benefits of adaptations that result from historical association with humans (e.g., tameness or neophilia). In less disturbed settings, there are many additional mechanisms that can, in principle, facilitate successful feralization (see Figure 1). Altogether, thriving worldwide feral populations reveal clear and ubiquitous limits on native species' abilities to infiltrate all available contemporary niches.

## 7 ECO‐EVOLUTIONARY EFFECTS OF DOMESTICATION AND FERALIZATION

Domestication can profoundly impact native ecosystems through a number of mechanisms. Animal cultivation practices, for instance, introduce nutrients and antibiotics, promote land use conversion, and modulate global climates through feed and cover cropping and greenhouse gas emissions. The effects of agriculture on coupled ecosystems are well reviewed and organized in current literature; they would also exceed the scope of this review. Here, we instead focus on ways that feralization can modulate fitness of other organisms in invaded landscapes. While the effects of feral invasions are often explored in the conservation‐based literature, the unique evolutionary influences of domestication histories are often downplayed or ignored in this body of research (Henriksen, Gering, & Wright, 2018). We hope to encourage increased focus on this specialized topic for several reasons. First, as outlined in prior sections of our review, artificial selection histories can uniquely alter both the traits and evolutionary potential of feral organisms. Additionally, ongoing associations with humans create atypical invasion routes and opportunities for feralizing species. Finally, as we describe below, managing feral animals often also presents unique regulatory and social challenges.

Like other biotic invaders, feral taxa can influence important ecological processes in invaded habitats (Table 3). These effects are often revealed by exclusion experiments, which produce diverse, pronounced, and sometimes counterintuitive functional modulations of ecosystems (e.g., Beasley, Ditchkoff, Mayer, Smith, & Vercauteren, 2018). For example, feral pig activities in Hawaii create the most abundant and productive breeding habitats for introduced mosquitoes, which in turn spread lethal pathogens to endemic and introduced birds (Nogueira‐Filho, Nogueira, & Fragoso, 2009). And in Australia, there is mixed evidence that feral predators (e.g., dingoes) regulate feral mesopredators (e.g., cats), which in turn regulate populations of both endangered and introduced small mammals (Allen, Allen, & Leung, 2015; Newsome et al., 2017). Feral domestics can also spread disease to wild populations, potentially serving as reservoirs and/or agents of selection that modulate interactions between pathogens and wild hosts (e.g., Madhun et al., 2017).

**Table 3:** Ecological impacts of feral animals on invaded ecosystems

Ecological impact	Example from the literature
Feral animals as predator or prey	Feral cat predation is the leading cause of bird mortality in the United States (Loss, Will, & Marra, 2013). Feral cats are also prey for dingoes in Australia (see text), where they are also a significant source of bird mortality (Woinarski et al., 2017)
Feral animals compete with native taxa	Native ungulates in the Western United States avoid water sources when feral horses are present, though aggressive interactions are rare (Hall, Larsen, Knight, & McMillan, 2018)
Feral animals alter community structure	Many feral ungulates alter communities through grazing and trampling vegetation (e.g., sheep on Santa Cruz Island in California; Schuyler & Sterner, 2002), though the specific changes produced are sometimes surprising. In coastal salt marshes, for example, feral horse activity may drive reversions to Pleistocene community assemblages (Levin, Ellis, Petrik, & Hay, 2002). This type of pattern fuels ongoing debate over rewilding and management objectives for contemporary ecosystems (e.g., Rubenstein & Rubenstein, 2016)
Feral animals alter nutrient cycling	Extirpation of feral pigs in a Hawaiian ecosystem increased soil nutrient regeneration and nitrogen availability (Long et al., 2017)
Feral animals transmit disease	Free‐roaming dogs in Chilean urban areas are rarely vaccinated against nonhuman pathogens and can facilitate disease spread to native carnivores (e.g., Acosta‐Jamett, Cunningham, Bronsvoort, & Cleaveland, 2015). Zoonotic diseases, such as rabies, typhus, and toxoplasmosis, can also flow between feral animals and human populations

These multiplicative effects of feral taxa on invaded ecosystems make them logistically challenging to study. Further, managing feral animals can present unique and formidable regulatory obstacles. Many feral taxa are inherently appealing and charismatic to humans, which can hinder public support for efforts to limit or eradicate feral populations. This is best exemplified by feral cats, which are a leading source of mortality for wild birds and small mammals worldwide (Table 3), and which also spread diseases that affect both humans and wildlife. Well‐meaning humans often feed and advocate for feral cats and other animals, and staunchly oppose regulatory efforts. As a result of this enthusiasm, some feral animals are even afforded legal protections. Feral horses, for example, have a strong presence in the culture of the Western United States and are legally protected by the Wild and Free‐Roaming Horses and Burros Act of 1971 (Iraola, 2005). As these examples show, managing feral invaders requires accounting for public perception, as well as animals' cultural and legal significance. This is doubly important because moral conflicts between wildlife managers and local communities can hinder public support for other acts of conservation, for example, efforts to control nonferal invaders (Novoa, Dehnen‐Schmutz, Fried, & Vimercati, 2017).

In summary, feral animals have demonstrably diverse and complex effects on invaded ecosystems, and our ability to manage these effects is hampered by both scientific and social challenges. Further research focusing on feralization can better inform effective management and also enlighten public attitudes concerning feral animal biology and control. For instance, while monitoring is sometimes done to assess the effects of complete eradications (e.g., Brooke et al., 2018, Hill, Coetsee, & Sutherland, 2018), fewer studies have examined how fluctuations of feral population densities change the structure and function of invaded communities. This is an important gap because permanent eradication may be impossible and/or impractical in many habitats where feral animals have become, or will become, well established. Despite the global ubiquity of feral animals, we also have very limited understanding of how they modulate higher‐level ecosystem processes such as nutrient cycling. These effects are likely nontrivial, given the high densities and activity levels of many feral populations.

Further research into the unique eco‐evolutionary dynamics of feral populations is also needed; this work may be aided by studies of feral animal's wild relatives (Sandoval‐Castellanos, Wutke, Gonzalez‐Salazar, & Ludwig, 2017), but the unique genetic features of feral species, owing to their domesticated pasts, may lead to unexpected feedbacks between population dynamics and fitness. Finally, proper communication of the ecological impacts of feral species can alter public perception and facilitate structured decision‐making management that incorporates public values (Estévez, Anderson, Pizarro, & Burgman, 2015; Gregory & Keeney, 2002; Loyd & Devore, 2010). To do this, we need the public to have a clearer understanding of the distinguishing features and consequences of feralization.

## 8 APPLICATIONS TO MANAGEMENT AND CONSERVATION

The impacts of artificial selection reviewed within this article have important ramifications for both natural and cultivated systems. For example, half of wild Norwegian salmon populations show introgression from farmed counterparts; this affects their life histories and lifetime fitness, and can therefore adversely affect food production and conservation objectives (Glover et al., 2012). It is also unlikely that these effects can easily be reversed, because introgression has also decreased genetic differentiation among recipient wild populations. It is therefore likely that we have already lost, through introgression and feralization, genetic variants with unknowable utilities to the future of food security. While further losses should be prevented, it is also unclear how best to limit maladaptive gene flow from domestic or feral populations into wild ones. Baskett and Waples (2013) concluded that ongoing escapes of small numbers of domesticated immigrants are often most harmful to wild recipient populations (vs. sporadic influxes of larger sizes). Unfortunately, this observation suggests it is important to mitigate mechanisms of gene flow that are often the most difficult to anticipate, detect, and prevent. In some cases, breeding sterile animals in captive settings can circumvent unintended admixture, but this option must be weighed carefully against the unique logistical, ethical, and social challenges it presents. In the absence of engineered sterility, Baskett and Waples also offer two orthogonal approaches to mitigating maladaptive admixture between wild and domesticated taxa. The first approach maximizes the genetic distinctions between parapatric farmed and local (wild) gene pools. The rationale for this is to help purge outbred individuals or gene variants that escape into the local wild. The second approach keeps the two populations as similar as possible, in order to minimize fitness loads in intercrossed individuals. The authors outline various merits of both approaches, but also emphasize that their logistical difficulties necessitate weighing potential costs of management failure as well. We encourage any readers who are tasked with management or consulting to delve further into Basket and Waples' study (and work cited therein) for additional insights that are relevant to many focal systems.

In addition to this recommendation, we also encourage researchers and collection managers to recognize the value of accessioning samples and/or fitness‐related data from domestic and feral populations. Feral taxa have attracted comparatively little attention from evolutionary biologists (Henriksen et al., 2018); identifying the factors that influence their success or failure is an important first step toward effective long‐term management, but this work also requires analyses that span both space and time. This kind of work is underway in fisheries systems, but virtually absent from terrestrial studies of feral animals (Laikre et al. 2010). Finally, focused research and extension work emphasizing feralization's multiplicative costs to wild and agricultural ecosystems can bolster public support for control efforts. This will also encourage citizens to minimize animal escape and release, curtailing both accidental and intentional establishments of feral populations, and thereby minimizing their eco‐evolutionary footprint.

## 9 CONCLUDING REMARKS

At the outset of this review, we expected to find a relatively small body of literature concerning how artificial selection modulates fitness. We were surprised, however, by the wealth of relevant information on this subject. Still, like others before us (e.g., Hemmer, 1990), we found this information to be widely scattered among poorly integrated subdisciplines, including animal science, conservation biology, behavioral ecology, genetics, and evolution. Understandably, authors of pertinent articles have often sidelined or eschewed explicit consideration of artificial selection in order to focus on other implications of their work. Our chief hope for this review is that, by conceptually unifying studies of feralization and domestication, we will encourage increased contemplation and inquiry into the unique evolutionary legacies of artificial selection. Looking forward, we can also identify one ironic limitation of the growing literature: At the time of this writing, the very same technologies that provide heightened insight into artificially selected systems (e.g., genome sequencing and editing) are simultaneously transforming these systems. In the past, each major advancement to domestication practices (see Figure 1) has fundamentally changed human civilization. Now, the recent discovery of genome editing technologies finds us on the cusp of another major advancement—one that will surely alter the ecological and evolutionary processes we have explored in this review. Our final hope is that by reflecting on evolutionary aspects and impacts of artificially selected taxa, we will better prepare ourselves to observe, anticipate, and manage the changing nature of the organisms human most depend upon, and which have brought us this far.

## CONFLICT OF INTEREST

None declared.

## DATA ARCHIVING STATEMENT

This review does not continue any original or unpublished data.
